# Small heat shock proteins and biomolecular condensates

**DOI:** 10.1007/s00018-026-06216-y

**Published:** 2026-05-01

**Authors:** Samuele Crotti, Valentina Secco, Marialaura Morini, Serena Carra

**Affiliations:** Department of Biomedical, Metabolic and Neural Sciences, University of Modena and Reggio Emilia, Modena, Italy

**Keywords:** Small heat shock proteins, Molecular chaperones, Liquid-liquid phase separation, Biomolecular condensates, Protein quality control.

## Abstract

Proteins comprise well-ordered structural domains and intrinsically disordered regions that explore broad conformational ensembles, a pervasive feature of the human proteome that underlies key aspects of cellular physiology. In the crowded intracellular environment, stress can shift protein conformational equilibria toward aggregation-prone states, exposing hydrophobic regions that can drive aberrant protein-protein interactions, promoting aggregation. To maintain proteome integrity, cells depend on an integrated protein‑quality‑control network in which molecular chaperones, their co‑factors and dedicated degradation systems act in concert. Within this network, small heat shock proteins serve as an ATP-independent first line of defense that stabilizes non-native proteins and limits irreversible aggregation. Recent work shows that small heat shock proteins can also safeguard the liquid‑like dynamics of biomolecular condensates formed by liquid-liquid phase separation. These membraneless compartments organize cellular biochemistry but are susceptible to stress- and disease-induced arrest or aggregation. Rather than undergoing phase separation autonomously, small heat shock proteins can be recruited into pre-existing condensates such as stress granules, nuclear speckles, p62 bodies, and condensates formed by disease-associated proteins, where they help preserve condensate fluidity. Together, these findings position small heat shock proteins as modulators of condensate dynamics that link protein quality control to mesoscale cellular organization, with important implications for cell biology, aging, and human disease.

**Graphical Abstract:**

**Figure:**
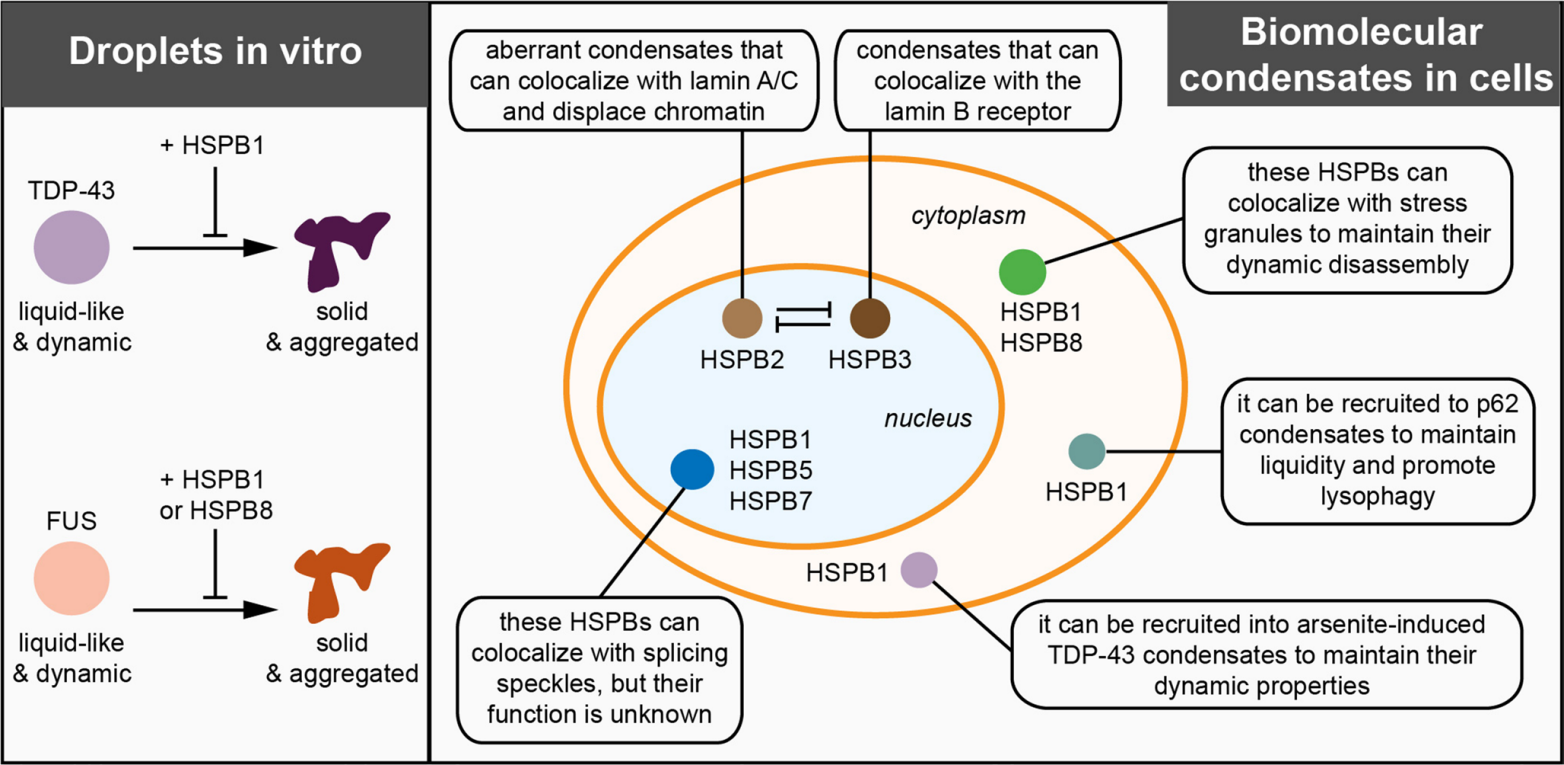
Summary of current evidence obtained in vitro and in cells about HSPB recruitment into droplets or biomolecular condensates and their putative roles

## The small heat shock proteins and the protein quality control system

Proteins can be classified based on their structural properties into three main groups: ordered (structured) proteins, intrinsically disordered proteins (IDPs), and proteins that are largely structured but contain intrinsically disordered regions (IDRs) [1–3]. Fully ordered proteins require folding into a defined three-dimensional conformation to achieve functional activity, whereas IDPs are biologically active without a fixed structure. It is estimated that roughly half of human proteins contain extensive IDRs and that fully disordered proteins represent 10–30% of the proteome [2–4]. The intracellular environment is highly crowded, with a cytosolic concentration reaching 300–400 g/L of proteins and other macromolecules [5, 6]. While crowding favors functional interactions, it also limits the entropic freedom of non-native polypeptides, potentially enhancing the likelihood of protein misfolding and aggregation [7–9]. Protein aggregation is a multistep process in which partially folded monomers, including IDPs, assemble into oligomeric intermediates via primary nucleation. These oligomers undergo structural conversion and maturation to form amyloid fibrils, which subsequently grow by monomer addition at fibril ends. In parallel, fibril surfaces catalyze secondary nucleation, generating new oligomeric species that further accelerate aggregation [10–12]. Aggregation is thermodynamically favored under supersaturation conditions, when protein concentration exceeds solubility [11, 13]. Of note, many proteins are expressed near their supersaturation limit and are, thus, particularly susceptible to aggregation [11, 14–16]. In addition, proteotoxic stress, such as oxidative conditions or heat shock, promotes protein denaturation and the exposure of normally buried hydrophobic residues; this, in turn, promotes misfolding, promiscuous protein-protein interactions and aggregate formation [8, 12, 17, 18].

To counteract misfolding and aggregation and maintain a healthy proteome, which is essential for life, cells have evolved a sophisticated Protein Quality Control (PQC) system, which consists of molecular chaperones and degradation systems [18–20]. Molecular chaperones interact with, stabilize, or assist other proteins to achieve their native conformation, without being part of the final folded protein [21–25]. They support de novo folding, refolding of denatured proteins, assembly of multimeric complexes, protein transport, and degradation, thus preserving proteome integrity [21–25]. Most molecular chaperones belong to the heat shock protein (HSP) superfamily, first identified as being upregulated in response to heat shock and other stressors, such as oxidative stress or viral infection [26, 27]. HSPs are highly conserved and were historically classified based on their molecular weight: Hsp100 (100–110 kDa), Hsp90 (90 kDa), Hsp70 (70 kDa), Hsp60 (60 kDa), Hsp40 (40 kDa) and small HSPs (with an average molecular weight < circa 35 kDa). Current nomenclature groups human HSPs based on sequence homology into six families: HSPA (Hsp70), HSPH (Hsp110), HSPC (Hsp90), DNAJ (Hsp40), HSPB (mammalian small HSPs), and human chaperonins (HSPD/Hsp60, HSPE/Hsp10, CCT) [28].

HSP expression is controlled by heat shock transcription factors (HSFs: HSF1-5, HSFX, HSFY) [29–31]. HSF1, the best-studied HSF, is kept in an inactive state in the cytoplasm through dynamic interactions with HSPA, HSPC and their DNAJ co‑chaperones, which prevent HSF1 trimerization and DNA binding [32]. Upon proteotoxic stress, HSPs preferentially bind misfolded substrates, releasing HSF1 monomers, which trimerize, translocate to the nucleus, and activate transcription of genes coding for HSPs and other heat shock response-related genes [29, 33]. HSF1 is then gradually downregulated via a negative feedback loop, involving HSP70/HSP40/HSC70 binding to its transactivation domain and eventually removing HSF1 from DNA [32]. HSF activity is further modulated by post-translational modifications (PTMs) such as phosphorylation, SUMOylation, acetylation, and ubiquitination [32]. Other HSFs display tissue-specific expression and regulate HSPs under physiological conditions, contributing to developmental processes such as embryogenesis, corticogenesis, and spermatogenesis [30, 31, 34].

Chaperone activity can be either dependent or independent on ATP hydrolysis. The best characterized function of ATP-dependent chaperones (which include members of the Hsp70/HSPA, Hsp90/HSPC, and Hsp60/HSPD families) is to promote folding or refolding of substrates, a process referred to as foldase activity, or assist client maturation (a function well-characterized for Hsp90/HSPC) [23, 35–37]. ATP-dependent chaperones can also target irreversibly misfolded proteins for proteasomal degradation or autophagic clearance [24, 38–40], often acting in concert with co-chaperones such as DNAJs and nucleotide exchange factors of the HSPH (Hsp110) family, which regulate the Hsp70 ATPase cycle and fine tune substrate binding and release [23, 35, 37, 41]. For a detailed description of ATP-dependent chaperone functions and mechanisms please refer to these reviews [22–25, 35–37]. By contrast, small HSPs (sHSPs) are ATP-independent. sHSPs, which are found in all kingdoms of life (bacteria, archaea, and eukarya), can recognize exposed hydrophobic residues on early unfolding intermediates, stabilizing them in a folding-competent state to facilitate subsequent refolding by ATP-dependent chaperones; this function is often referred to as holdase function [42–46]. Emerging evidence indicates that sHSPs (referred to as HSPBs in mammals) can also interact with natively folded proteins, regulating their assembly into multimeric complexes or preventing early-stage aggregation without disrupting native folding [47–50].

**Fig. 1:**
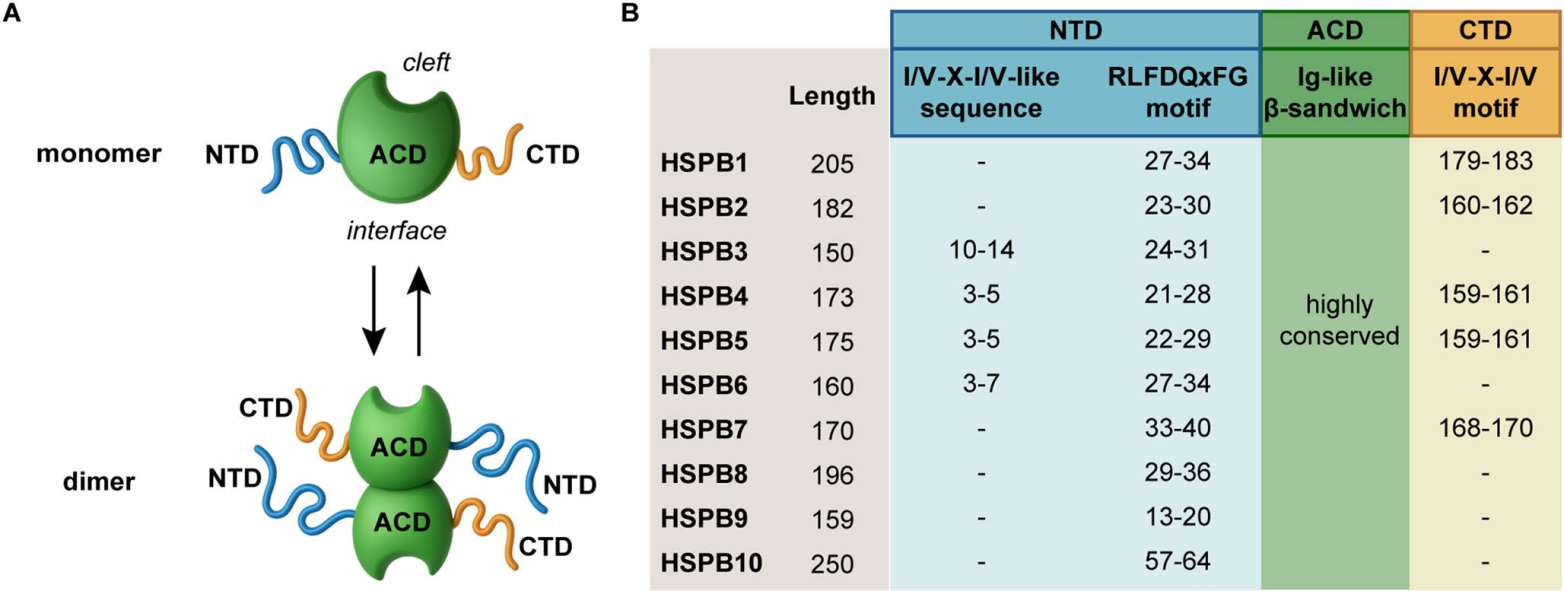
Schematic of sHSP/HSPB architecture. (**A**) Simplified representation of sHSP/HSPB structural organization. The upper panel shows a generic sHSP/HSPB monomer which contains a disordered hydrophobic NTD (blue), a conserved ACD (green) forming an Ig-like β-sandwich, and a disordered polar CTD (yellow). The ACD presents a dimer interface which mediates sHSP/HSPB dimerization (lower panel), and a hydrophobic cleft which is involved in client protein binding and in the formation of oligomers. The cartoon graphic was edited using Microsoft Copilot. (**B**) Schematic representation of the structural elements of human HSPBs showing the conserved ACD (green) flanked by the variable NTD (blue) and CTD (yellow). The table shows for the ten human HSPBs: - the protein sequence length; - the position of the I/V-X-I/V-like motif located in the NTD; - the position of the conserved RLFDQxFG motif located in the NTD; - the position of the I/V-X-I/V motif in the CTD

Structurally, sHSPs across all life are characterized by a low molecular weight (12–43 kDa), and a conserved alpha-crystallin domain (ACD), flanked by variable N-terminal (NTD) and C-terminal (CTD) domains (Fig. 1A) [51, 52]. The ACD consists of 90–100 residues forming an immunoglobulin-like β-sandwich with antiparallel β-sheets [44, 52–54]. The anti-parallel alignment of specific ACDs’ β-strands form a β-sheet dimer interface which mediates sHSP dimerization (Fig. 1A) and can be involved in substrate binding [50, 52–54]. The NTD and CTD, which can represent more than 50% of the protein sequence, are intrinsically disordered [44, 53, 55]. The NTD, which ranges from 24 to 247 residues depending on the species (typically 50–100 residues in vertebrates), is enriched in hydrophobic residues and phosphorylation sites, and contains a core RLFDQxFG motif (Fig. 1B) [51, 52, 56]. The CTD can contain another typical motif called the I/V-X-I/V motif, which participates in the process of sHSP oligomerization, while I/V-X-I/V-like sequences can be found in the NTD (Fig. 1B) [44, 52–54]. Both NTD and CTD regulate the assembly of sHSPs into dynamic homo- or hetero-oligomers, ranging from dimers to approximately 40–50mers (up to ~ 28–30mers for HSPB1 and HSPB5) [52, 53, 57, 58]. In agreement, human HSPB3, HSPB6 and HSPB8 lack the C-terminal I/V-X-I/V motif and therefore display a reduced ability to form medium-to-large oligomers [53]. The association and dissociation of sHSPs in homo- or hetero-oligomers is modulated by pH, temperature, or PTMs, especially by phosphorylation, and can influence both binding affinity to substrates and chaperone activity [44, 53], 59– [61]. In addition to confer high structural flexibility, the NTD and CTD of sHSPs regulate their interaction with diverse substrates (referred to as binding plasticity) [50, 53], 62– [65]. Multiple binding sites in the ACD, NTD, and CTD can participate in the interaction with substrates, and the contribution of specific domains differs depending on substrate identity and conformational state [44, 49, 50, 60, 66, 67].

Thanks to their ability to interact with a large variety of substrates, sHSPs perform pleiotropic roles and have been suggested to indirectly regulate complex processes ranging from apoptosis to cytoskeletal dynamics and cell differentiation [63, 68–70]. Finally, the structural flexibility of sHSPs’ NTD and CTD promote dynamic multivalent interactions that can drive liquid-liquid phase separation (LLPS). The latter is a process that enables protein compartmentalization in space and time, generating specialized microenvironments, referred to as biomolecular condensates, that participate in the regulation of a large variety of cellular processes [71, 72]. Several studies have identified sHSPs as components of biomolecular condensates in diverse cellular contexts and across different model organisms. The following sections describe the principles of LLPS, the cellular functions of phase-separated compartments, and summarize current knowledge on sHSPs and their recruitment into various condensates. Overall, the experimental data suggest that, by sampling broad oligomeric ensembles and engaging clients through multivalent, reversible interactions, sHSPs are ideally positioned to continuously reshape the protein conformational landscapes, rather than only passively suppressing aggregation, helping to keep condensates away from aberrant conversion into an aggregated-like state that has been associated with dysfunction and disease.

## Principles of LLPS and biomolecular condensates

Cellular compartmentalization is essential for the spatiotemporal regulation of biochemical processes [72]. Cellular compartmentalization is achieved through membrane-bound organelles, such as the nucleus, mitochondria, endoplasmic reticulum (ER) and Golgi apparatus, which are enclosed by lipid bilayers that physically separate their contents from the cytoplasm and block the passive diffusion of macromolecules [72]. In addition to compartments defined by a lipid‑based barrier, cells also employ membraneless organelles, or biomolecular condensates, which arise through selective concentration of proteins, RNA and other macromolecules, to support specialized cellular functions such as the enhancement or inhibition of biochemical processes, the buffering of protein concentrations, the sensing of changes in the environment and the exertion of mechanical forces [72–75]. As mentioned above, condensates form through LLPS, a biophysical process driven by multivalent, weak interactions that allow macromolecules to demix from the surrounding cytoplasm and assemble into dense and dynamic phases [72–76]. Thermodynamic principles underlying LLPS and models describing how multivalent interactions govern condensate formation have been comprehensively addressed in previous works [77–80]. Importantly, the propensity of a system to undergo phase separation is highly sensitive to the concentrations and intrinsic properties of each macromolecule, as well as to environmental conditions such as temperature, salt composition, ionic strength, pH, and macromolecular crowding. Consequently, many macromolecules exhibit phase behaviors that are responsive to diverse physicochemical stimuli [72, 74, 76].

But what are the molecular features driving LLPS? Multivalency refers to the ability of a molecule to engage in multiple, simultaneous, and reversible interactions and is central to LLPS [72, 74, 75, 81]. IDRs or repeated modular domains provide such multivalent interactions. IDRs lack stable tertiary structures and exist as ensembles of conformations, enabling flexible binding interfaces [82, 83]. IDRs involved in condensate formation often contain distributed short interaction motifs that, through particular residue chemistries (aromatic, charged, polar, hydrophobic), mediate weak multivalent interactions, which collectively drive phase separation and can impart some selectivity. This mode of assembly has been conceptualized with the model of stickers and spacers, where the interaction motifs are thought to function as cohesive stickers that crosslink neighboring proteins through weak interactions, while spacer regions provide conformational flexibility, promoting the liquid-like behavior of the dense phase [77, 84]. Several different classes of phase-separating IDRs can be classified based on their sequence compositions and charge distribution: these regions often contain low-complexity sequences, prion-like domains (PrLDs), or clusters of charged/aromatic residues, which facilitate transient interactions through cation–π, π–π stacking, electrostatic interactions, dipole-dipole, and hydrogen bonding [77, 85, 86]. In contrast, repeated modular domains, such as SH3, WW, PDZ, or RNA recognition motifs (RRMs), are folded structures that interact with complementary motifs on ligands, enabling proteins to establish a multivalent interaction network [72, 81, 83, 87]. Because each domain binds a specific ligand (e.g. RNA, proline-rich motifs, peptides or other proteins), modular domains also determine the molecular composition and selectivity of condensates.

In addition to proteins, RNA has been identified as a key component that drives condensate formation and shapes its biological function [88]. The interaction with RNA enhances the ability of several RNA-binding proteins (RBPs) to phase separate by lowering the concentration threshold for droplet formation (saturation concentration) and increasing the interaction valency [88–90]. Depending on its length and structure, RNA can also tune the material properties of condensates and can selectively recruit specific proteins into RNP condensates [88].

Thus, condensates are heterogeneous molecular assemblies composed of thousands of components functioning as either scaffolds or clients [91]. Scaffolds are highly multivalent molecules that drive condensate assembly and influence the saturation concentration (c_sat) at which phase separation occurs. A scaffold protein is typically a large, abundant, non-enzymatic molecule whose absence disrupts condensate formation [71]. The Ras GTPase-activating protein-binding protein G3BP1 and the homologous protein G3BP2 exemplify this role: they are abundant cytosolic proteins that act as core structural components of stress granules (SGs) when during stress cytoplasmic mRNA is released from polysomes [92, 93]. By contrast, clients possess lower interaction valences and are neither necessary nor sufficient to drive phase separation, but they are recruited to condensates that are formed by scaffolds [72]. Clients can include enzymes, signaling molecules, or RNAs and their recruitment is thought to modulate their activity and availability. This scaffold-client architecture, and the formation of internal subdomains within each condensate, enables spatial segregation of biochemical reactions and rapid, reversible regulation of processes that range from 3D genome organization to chromatin architecture, transcription, ribosome biogenesis and signal transduction [72, 94–96].

Based on their timing of formation within cells, biomolecular condensates can be categorized as ubiquitous or stress inducible. Ubiquitous condensates, such as the nucleolus, nuclear speckles and membrane-interacting condensates, are constitutively present in cells and participate in fundamental processes such as ribosome biogenesis, mRNA maturation or transmembrane signaling [97–99]; in contrast, stress-inducible condensates, including SGs, DNA repair condensates, NELF condensates, assemble transiently in response to stress conditions, such as heat shock, oxidative stress, or nutrient deprivation that are accompanied by translation and transcription attenuation [100–103]. The list of condensates that differ for composition and functions is rapidly growing. To the already listed examples, we also cite: chromatin condensates, such as heterochromatin domains, that partition the genome into transcriptionally active and inactive regions [104]; transcriptional and enhancer condensates, that concentrate the transcription machinery at specific loci to increase gene expression efficiency [105, 106]; splicing condensates, including nuclear speckles, paraspeckles, and Cajal bodies, that coordinate assembly of splicing machinery and RNA processing [98, 107, 108]. In the cytoplasm, several condensates act as RNA storage and triage sites: RNA transport granules shuttle transcripts to precise cellular destinations; SGs transiently sequester mRNAs during environmental stress likely to protect them from degradation; processing bodies (P-bodies) serve as hubs for mRNA decay [100, 101]. Lately, a subset of RNA granules has been proposed to arise as “incidental condensates”, structures that form simply when RNP complexes reach sufficiently high local concentrations, without necessarily carrying out any dedicated biological function [109].

In the following sections, we will focus on the current literature describing the recruitment of sHSPs into condensates across different organisms (human and yeast) and their putative roles within these compartments.

## Mammalian HSPBs

**Fig. 2:**
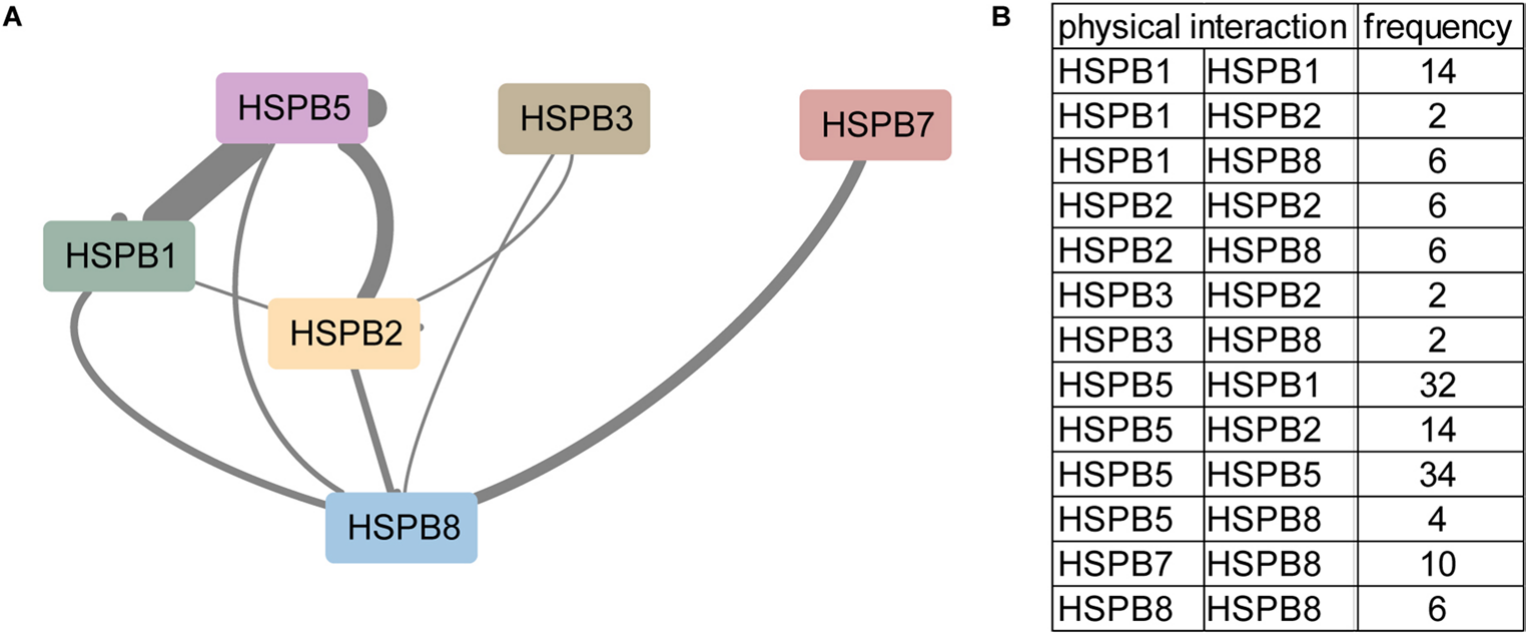
Interaction network among HSPB family members. (**A**) Interaction network of HSPB1, HSPB2, HSPB3, HSPB5, HSPB7, and HSPB8, constructed using physical interaction data from BioGRID (v5.0.253, January 2026) and visualized in Cytoscape. Edge thickness is proportional to the number of independent experiments supporting each interaction. (**B**) Number of experimentally supported physical interactions for each HSPB pair as reported in BioGRID

The human genome contains 10 genes coding for small HSPs that according to the new nomenclature are referred to as HSPB1 – HSPB10 (Fig. 1B) [28]. The best characterized members of the family include HSPB1, HSPB4, HSPB5 and HSPB8; instead, the other members have been less-well characterized, and their biological functions and exact mechanisms of action are still largely unknown [110]. A number of experimental evidence in vitro and in cells documented the colocalization of HSPB1 and HSPB8 with proteins that either undergo phase separation or are recruited inside biomolecular condensates [111–117]. In parallel, additional studies indicate that HSPB2 and HSPB3 can assemble into liquid‑like, dynamic condensates within cells [118–120]. Furthermore, HSPB1, HSPB5 and HSPB7 have been reported to colocalize with nuclear condensates, although their functional roles in this context remain unresolved [121–128]. We provide a graphical overview of the physical interactions between HSPB1, HSPB2, HSPB3, HSPB5, HSPB7 and HSPB8 according to BioGRID (Fig. 2A), as well as their frequency (Fig. 2B). Importantly, these physical interactions are based on experiments performed in vitro, with recombinant HSPBs and/or in cell lysates. While some interactions have been confirmed both in vitro and in cells, such as for example the HSPB1 and HSPB5 hetero-oligomeric complexes [129, 130] or the HSPB2 and HSPB3 hetero-oligomeric complexes, with fixed 3:1 stoichiometry [131–133], the interaction between HSPB7 and HSPB8 has been observed in vitro [134]. Instead, in cells HSPB7 and HSPB8 interact with other specific partners such as Filamin C [47, 135] or the Hsp70 co-chaperone BAG3 [133, 136, 137], respectively.

### HSPB1

Also known as Hsp27, HSPB1 is a 23 kDa protein constitutively and widely expressed across human tissues with the highest expression in skeletal, smooth, and cardiac muscles [63]. It performs multiple essential cellular functions, including chaperone activity to prevent protein misfolding and aggregation [138–143], regulation of cytoskeletal organization to ensure proper assembly and cell motility [144–147], anti-apoptotic activity through interactions with both pro- and anti-apoptotic factors [148–150], and modulation of cellular redox balance [151]. HSPB1 forms dynamic oligomers whose size and composition are tightly regulated by phosphorylation at the key serine residues S15, S78, and S82 [146, 152–155]. In its non-phosphorylated state, HSPB1 assembles into large homo-oligomeric complexes consisting of more than 20 subunits [156]. Besides being able to form homo-oligomers, HSPB1 can interact with other HSPBs (e.g. HSPB5 and HSPB6) forming hetero-oligomeric complexes with distinct properties [130, 157, 158]. Phosphorylation triggers dissociation of these large oligomers into smaller species, which generally exhibit enhanced chaperone activity [156, 159, 160]. HSPB1 phosphorylation is primarily mediated by MAPK-activated protein kinase 2 (MK2), activated downstream of p38 MAPK during stress [161]. Other kinases, including MK5-PRAK, PKCγ, and PKD, also target these serine residues [161]. These phosphorylation-dependent structural rearrangements enable HSPB1 to adopt different oligomeric states, influencing subcellular localization, cytoskeletal interactions, and recruitment to phase-separated compartments [111–115, 121, 122, 162–164].

**Fig. 3:**
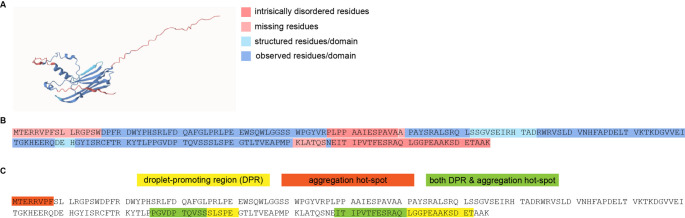
Predicted 3D structural model and LLPS potential of HSPB1. The 3D structural model of HSPB1 was generated using AlphaFold (**A**). (**B**) Its intrinsically disordered residues were predicted using MobiDB (https://mobidb.org/). (**C**) HSPB1 droplet-promoting regions and aggregation hot-spot residues according to FuzDrop (https://fuzdrop.bio.unipd.it/predictor)

A structural and biophysical overview of HSPB1 is provided in Fig. 3.

#### HSPB1 and RBP condensates

In vitro studies indicate that HSPB1 does not phase separate autonomously [112]. Instead, it is selectively recruited into condensates formed by RBPs such as FUS and TDP-43 [111, 112]. Both RBPs undergo LLPS to form condensates that can mature over time from a liquid-like state into gel- or solid-like states, a process that facilitates amyloid formation [90, 165, 166]. This process, referred to as “condensate aging”, is accelerated by disease-associated mutations in FUS and TDP-43 and is thought to contribute to the formation of the pathological aggregates observed in amyotrophic lateral sclerosis (ALS) and frontotemporal dementia (FTD) [165, 166]. FUS aggregates occur in ~ 10% of FTD and ALS cases, whereas TDP-43 inclusions are a hallmark in the majority of these patients and are also detected in ~ 50% of Alzheimer’s disease cases, as well as in Parkinson’s disease (PD), Huntington’s disease (HD), and limbic-predominant age-related TDP-43 encephalopathy (LATE) [167–169]. Recruitment of HSPB1 into FUS and TDP-43 condensates has been documented both in vitro and in cells [111–113]. Concerning FUS, in vitro studies showed that HSPB1 binds directly to the low-complexity domain (LC) of FUS, reducing its self-assembly into liquid-like droplets [111]. This inhibitory activity requires the HSPB1 NTD and it is further boosted by its multimerization [111]. Phosphorylation at S15, S78, and S82 induces a functional switch of HSPB1: it weakens its ability to inhibit FUS LLPS while enhancing its activity in modulating amyloid aggregation. Indeed, phosphorylation diminishes the interaction between the HSPB1 NTD and the FUS LC, as well as the multimeric assembly of HSPB1, thereby diminishing its inhibitory effect on FUS LLPS. In contrast, phospho-mimetic HSPB1 mutants (3D, S15D/S78D/S82D) partition more efficiently into FUS droplets compared to wild-type HSPB1, contributing to maintain droplet liquidity and prevent amyloid formation [111]. Domain mapping, NMR spectroscopy and computational modelling revealed that accessible HSPB1 NTDs are essential for both condensate partitioning and client binding [111, 170]. Concerning TDP-43, in vitro HSPB1 binds both the LC and RRM1 domains of TDP-43 and partitions into TDP-43 droplets, delaying droplet maturation into gel- or solid-like states [112]. Cellular studies using APEX proximity labelling, quantitative mass spectrometry, immunofluorescence and FRAP showed that HSPB1 binds to cytoplasmic ΔNLS-TDP-43 (lacking the nuclear localization signal/NLS) and colocalizes in phase separated cytoplasmic condensates, thereby preventing ΔNLS-TDP-43 protein immobilization [112]. Of note, these cytoplasmic ΔNLS-TDP-43 condensates that colocalized with HSPB1 were independent of SGs and converted into an aggregated state in absence of HSPB1 [112]. It is important to mention that the oxidative stress conditions that induce the cytoplasmic aggregation of TDP-43 lead also to the formation of SGs. SGs are RNA-protein condensates that form upon polyribosome disassembly, transiently storing non-translating mRNAs together with RBPs such as TDP-43 and FUS, and other translation-repressing factors [100, 171]. Upon stress relief, SGs disassemble, releasing RNA back into the translational pool [101]. Although TDP-43 aggregation can occur independently of SGs [172], a fraction of the protein is recruited inside arsenite-induced SGs, where it can undergo de-mixing and aggregation [166]. Thus, both direct TDP-43 aggregation in the cytoplasm and recruitment of TDP-43 inside SGs, followed by up-concentration and aggregation within SGs have been documented and are thought to contribute to the formation of the inclusion bodies during the course of disease [166, 172]. Interestingly, HSPB1 colocalizes with cytoplasmic SGs in multiple cell types, opening the possibility that it could also help to prevent TDP-43 aggregation within SGs [111–113, 116, 173]. The conversion of SGs from a dynamic into an aggregated state has been repeatedly documented. Although misfolded proteins such as defective ribosomal products (DRiPs) tend to be compartmentalized in specific quality control compartments, both in the cytoplasm and the nucleus [174], they can also end up inside SGs [116]. This, in turn can promote the conversion of SGs from a dynamic state into a solid-like arrested state, a process that can be countered by several molecular chaperones and co-chaperones, including VCP, HSP70, BAG3 and HSPB8 (see later) [113, 116, 175]. Importantly, HSPB1 is recruited specifically into SGs that contain misfolded proteins such as DRiPs, but also misfolded Ubc9-TS and mutant SOD1-G93A [113, 116, 176]. Quantitative imaging revealed that HSPB1 is absent from newly formed SGs lacking misfolded proteins, but it is progressively recruited as misfolded proteins accumulate [113]. In line with these observations, HSPB1 knockdown correlates with accumulation of SGs enriched in misfolded proteins, which also display a delayed disassembly [112, 116]. Collectively these observations show that HSPB1 can be recruited inside condensates containing RBPs such as TDP-43 and FUS, as well as cytoplasmic SGs that become enriched for misfolded proteins, where it helps to maintain protein mobility and liquid-like condensate properties.

#### HSPB1, p62 condensates and lysophagy

Beyond RNA-protein condensates, HSPB1 plays an emerging role in selective autophagy by regulating p62/SQSTM1 condensates during the clearance of dysfunctional lysosomes (lysophagy) [114, 177]. Of note, lysophagy impairment is linked to neurodegenerative diseases, including PD, AD, ALS and FTD [178, 179]. Lysophagy depends on ubiquitin-mediated recruitment of adaptor proteins, including p62/SQSTM1 and NBR1 [180, 181]. p62 and NBR1 function as a scaffold by binding to the ubiquitinated cargo and to LC3 on forming autophagosomes [182]. Recombinant p62 forms condensates only when engaged with K63-linked polyubiquitin chains [183], and this process is regulated by ubiquitin interactions, self-oligomerization and phosphorylation [183, 184]. Upon lysosomal damage, p62 condensates incorporate ubiquitinated proteins and autophagic machinery and also recruit phosphorylated HSPB1 [114, 185]. HSPB1 interacts with p62 via its PB1 domain, and disruption of this domain impairs p62 localization and HSPB1 puncta formation following lysosomal damage [114, 186]. Within these condensates, phosphorylated HSPB1 maintains liquid-like properties that would facilitate autophagosome formation and efficient engulfment and clearance of damaged lysosomes. In agreement with this idea, loss of HSPB1 impairs condensate dynamics, inhibits lysophagy initiation and reduces autophagic turnover of damaged lysosomes [114].

In summary, rather than forming condensates itself, HSPB1 partitions into SGs, RBP condensates and p62 bodies to help preserve their dynamic, liquid-like state.

### HSPB2 and HSPB3

HSPB2 (a 20.2 kDa protein also known as MKBP) and HSPB3 (a 17 kDa protein also known as HSPL27) are two functionally related HSPBs that assemble into a well-defined hetero-oligomeric complex with a fixed 3:1 HSPB2:HSPB3 subunit ratio [131, 187]. The interaction was also observed in vivo [132]. In vitro, HSPB2 forms dynamic, low-molecular weight oligomers, predominantly hexamers or octamers, in a concentration-dependent manner [188, 189]. In contrast, the oligomeric behavior of HSPB3 is less well defined. While some studies suggest that HSPB3 does not engage in homotypic interactions and forms only small oligomeric species, ranging from dimers to tetramers, more recent work has reported the formation of heterogeneous oligomers spanning a wide size range, from dimers up to 30-mers [132, 189, 190]. Functionally, HSPB2 exhibits holdase activity and can only mildly prevent aggregation of misfolded proteins in vitro, depending on the substrate [188, 189, 191]. The chaperone activity of HSPB3 is also relatively weak and restricted to a limited set of substrates [189]; this poor chaperone activity seems to depend on the absence of the CTD, a structural feature that contributes to client binding in other HSPBs [189, 190]. In cells, HSPB3 does not confer thermotolerance, whereas HSPB2 enhances cell survival following heat stress [131, 192]. Similar to the proteins expressed alone, also the HSPB2-HSPB3 hetero-oligomers display moderate chaperone-like activity toward selected substrates, both in vitro and in cellular systems [131, 132, 143, 193]. Together these findings suggest that these proteins may act as specialized chaperones. In humans, HSPB2 and HSPB3 are predominantly expressed in cardiac and skeletal muscle tissues [132, 194–197]. In addition, HSPB3 expression has been detected in the brain, peripheral motor neurons, and fetal tissues [198, 199]. Unlike many other HSPBs, HSPB2 and HSPB3 are not induced by heat shock. Instead, their expression is transcriptionally upregulated by the myogenic transcription factor MYOD1 during myoblast differentiation into myotubes [119, 132].

Interestingly, during myogenic differentiation, HSPB2 and HSPB3 form distinct nuclear and cytoplasmic foci reminiscent of phase-separated condensates [118, 119]. Nuclear condensates that undergo fusion upon contact to form larger droplets and that display rapid exchange of HSPB2 and HSPB3 molecules with the surrounding nucleoplasm have likewise been observed in mammalian cells overexpressing HSPB2 and HSPB3 [118, 119]. The formation of nuclear condensates depends on IDRs of these HSPBs, specifically the CTD of HSPB2 and the NTD of HSPB3 [118–120]. Concerning the function of these nuclear condensates, experimental data suggest that they may influence nuclear lamin distribution and chromatin remodeling [118, 119]. HSPB2-containing nuclear compartments sequester the nuclear intermediate filament protein lamin A/C (LMNA), thereby altering its nuclear distribution and mobility and locally influencing chromatin organization [118]. Consistently, proximity-labelling experiments revealed a marked depletion of chromatin-associated proteins, histones, and DNA from HSPB2 condensates [120]. Importantly, the ability of HSPB2 to form intranuclear compartments is suppressed by HSPB3 [118, 120]. Conversely, increasing HSPB3 levels promotes the formation of static, irregularly shaped nuclear foci that sequester both HSPB2 and HSPB3 [119]. These HSPB2-HSPB3 assemblies additionally recruit other IDPs, as well as factors involved in chaperone-assisted protein folding and autophagy, suggesting that they can be targeted for clearance. Remarkably, these assemblies are reversible, as shifting the stoichiometry toward HSPB2 leads to their dissolution [120]. Thus, the relative abundance of HSPB2 and HSPB3 appears to be a critical determinant of their subcellular localization, dynamic properties and interaction with cellular components.

HSPB3 nuclear condensates also colocalize with the lamin B receptor (LBR) and can promote its relocalization from the nuclear envelope to the nucleoplasm [119]. In undifferentiated cells, LBR interacts with lamin B1 (LMNB1) and heterochromatin protein 1 (HP1), thereby tethering peripheral heterochromatin to the nuclear envelope and repressing the expression of pro-differentiation genes, including myogenic genes [200, 201]. During myoblast differentiation, this LBR-based tether is replaced by a lamin A/C–dependent tether, leading to chromatin reorganization and activation of muscle-specific transcriptional programs [201]. Notably, depletion of HSPB3 in differentiated myoblasts prevents this tether switch, resulting in impaired expression of myogenic and pro-differentiation genes [119]. Conversely, HSPB3 overexpression drives LBR sequestration into nuclear condensates, reducing its association with the nuclear envelope; this, in turn, could favor the LBR-LMNA tether switch, thereby promoting transcriptional changes associated with differentiation [119]. Co-expression of HSPB3 with HSPB2 reciprocally inhibits the formation of nuclear condensates that interact with either LBR or LMNA, highlighting the fine-tuned balance between these two HSPBs [118, 119]. Accordingly, modest local fluctuations in HSPB2 and HSPB3 expression levels, arising from their differential transcriptional regulation during differentiation, may be sufficient to drive the transient formation of HSPB3-rich condensates that contribute to promote the myogenic transcriptional program [119]. This interpretation is supported by the following findings. Two HSPB3 variants that disrupt the formation of the HSPB2-HSPB3 tetrameric complex, namely HSPB3-A33AfsX50 and HSPB3-R116P, have been identified in patients with congenital myopathy [118]. In cells, neither variant can prevent aberrant HSPB2 condensate formation: HSPB3-A33AfsX50 is unstable and rapidly degraded by the proteasome, whereas HSPB3-R116P fails to interact with HSPB2 and instead forms intranuclear aggregates [118]. These aggregates sequester both wild-type HSPB3 and LBR, leading to loss of HSPB3 function and immobilization of LBR, which can locally disrupts the LBR-LMNA tether switch, potentially compromising myoblast differentiation [119]. At present, the upstream signals that trigger HSPB2 and HSPB3 nuclear condensate formation in cells, beyond local concentration increases and the contribution of IDRs, remain unknown.

**Fig. 4:**
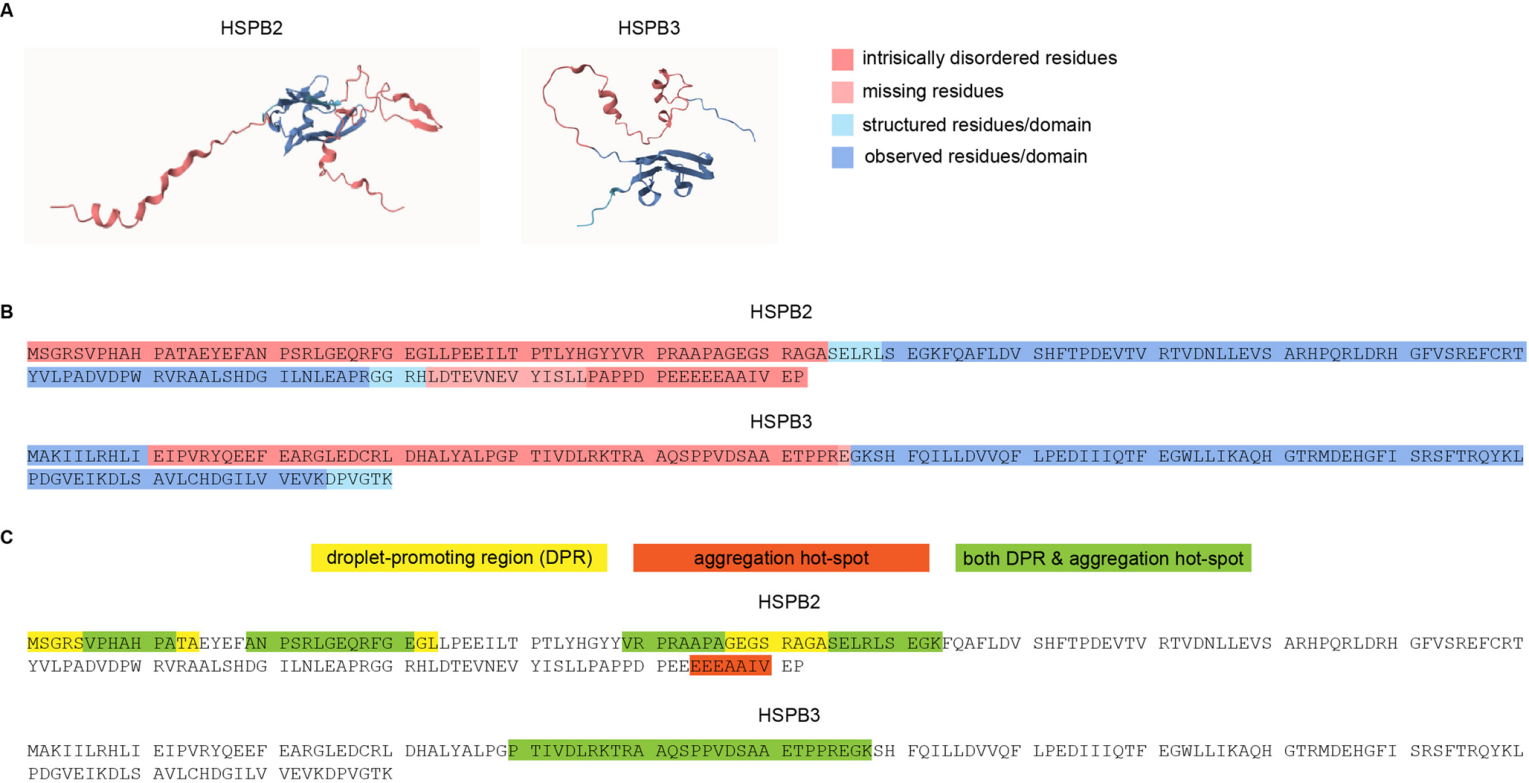
Predicted 3D structural model and LLPS potential of HSPB2 and HSPB3. The 3D structural model of HSPB2 and HSPB3 was generated using AlphaFold (**A**). (**B**) HSPB2 and HSPB3 intrinsically disordered residues predicted by MobiDB (https://mobidb.org/). (**C**) HSPB2 and HSPB3 droplet-promoting regions and aggregation hot-spot residues according to FuzDrop (https://fuzdrop.bio.unipd.it/predictor)

A structural and biophysical overview of HSPB2 and HSPB3 is provided in Fig. 4.

### HSPB5

Also known as αB-crystallin, HSPB5 is a 20 kDa protein highly expressed in the eye lens. It was originally considered lens-specific due to its role in preventing protein aggregation and maintaining lens transparency [202]. Subsequent studies revealed that HSPB5 is widely expressed in multiple tissues, including skeletal and cardiac muscle, brain and kidney, and its functions extend beyond lens maintenance [203]. HSPB5 exhibits broad chaperone activity, interacting with a variety of misfolded proteins to prevent their aggregation [141, 204–206]. It also modulates cytoskeletal organization through interactions with actin, intermediate filaments, and other cytoskeletal components [207–212]. Additionally, HSPB5 contributes to apoptosis regulation by inhibiting pro-apoptotic factors such as Bax, Bcl-Xs, and caspase-3 [213–216]. Structurally, HSPB5 exists as large, polydisperse oligomers comprising up to 40 subunits, which consist of homo- and/or hetero-oligomeric complexes with other HSPBs (e.g., HSPB1, HSPB4 and HSPB6) [53, 130, 217, 218]. Phosphorylation at three conserved serine residues in the NTD (S19, S45, and S59) induces dissociation of these large oligomers into smaller assemblies, a process linked to changes in chaperone activity [219–222]. HSPB5 phosphorylation can be triggered by diverse stimuli, including oxidative stress [223], arsenite stress and heat shock [222]. While the kinase responsible for the phosphorylation of S19 is still unknown, p44/42MAP kinase has been shown to phosphorylate S45 [224], whereas MAPKAP kinase-2 selectively phosphorylates S59 [224, 225].

**Fig. 5:**
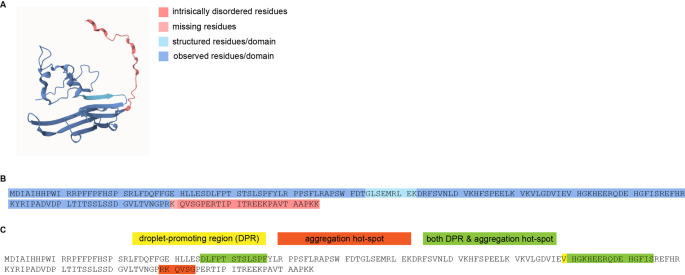
Predicted 3D structural model and LLPS potential of HSPB5. The 3D structural model of HSPB5 was generated using AlphaFold (**A**). (**B**) HSPB5 intrinsically disordered residues according to MobiDB (https://mobidb.org/). (**C**) HSPB5 droplet-promoting regions and aggregation hot-spot residues predicted by FuzDrop (https://fuzdrop.bio.unipd.it/predictor).

A structural and biophysical overview of HSPB5 is provided in Fig. 5.

Recent studies have explored the behavior of HSPB5 in phase-separated compartments in vitro and in cells [115, 123–125, 127, 128]. These include TDP-43 condensates in vitro and nuclear splicing speckles (for the latter see dedicated section below).

#### HSPB5 and TDP-43 condensates

Although there is no evidence that HSPB5 undergoes LLPS on its own, it can function as a regulator of TDP-43 phase separation [115]. According to recent in vitro findings, HSPB5 does not actively promote phase separation of TDP-43 LC under stress conditions, but it modulates and stabilizes TDP-43 droplets once they form [115]. Indeed, HSPB5 efficiently partitions into pre-formed TDP-43 LC condensates and delays their maturation into gel- or solid-like states by maintaining TDP-43 mobility [115]. To date, no cellular studies have been conducted to confirm these in vitro observations.

### HSPB7

Also known as cardiovascular HSP (cvHSP), HSPB7 is a 19 kDa protein predominantly expressed in cardiac and skeletal muscles [226]. Despite being relatively unexplored within the HSPB family, recent studies have begun to shed light on its structural and functional properties. Structurally, HSPB7 exists in equilibrium between large oligomers of approximately 600 kDa and smaller species around 36 kDa, likely corresponding to dimers [134]. This equilibrium is influenced by the formation of a disulfide bond involving cysteine 126 [134]. Oligomerization is further regulated by a unique N-terminal serine-rich region (residues 17–29), which is absent in other HSPBs [134]. Deletion of this polyserine stretch prevents formation of large oligomers [134]. From the structural point of view, HSPB7 interacts with the actin-binding protein filamin C (FLNC) and regulates its dimerization [47]. FLNC dimerization is promoted by phosphorylation at threonine 2677, whereas the formation of the HSPB7-FLNC heterodimer is induced under biomechanical stress and following phosphorylation of FLNC at tyrosine 2683 [47]. Taken together, the interaction of HSPB7 with FLNC seems to be critical for maintaining sarcomeric integrity, as skeletal-muscle-specific knockout of HSPB7 results in progressive diaphragm myopathy, with mislocalization and aggregation of FLNC [135]. Besides the specific interaction with FLNC, one of the best characterized functions of HSPB7 is its potent anti-aggregation activity toward proteins containing expanded CAG repeats encoding polyglutamine (polyQ) tracts, including huntingtin (HTT) [227].

**Fig. 6:**
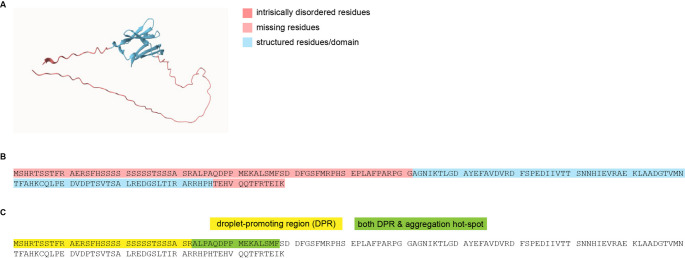
Predicted 3D structural model and LLPS potential of HSPB7. The 3D structural model of HSPB7 was generated using AlphaFold (**A**). (**B**) HSPB7 intrinsically disordered residues according to MobiDB (https://mobidb.org/). (**C**) HSPB7 droplet-promoting regions and aggregation hot-spot residues predicted by FuzDrop (https://fuzdrop.bio.unipd.it/predictor)

A structural and biophysical overview of HSPB7 is provided in Fig. 6.

#### HSPB7 and huntingtin condensates

Expansion of the CAG repeat in exon 1 of the HTT gene is associated with HD, a neurodegenerative disorder characterized by misfolding and aggregation of HTTex1 fragments [228]. HTTex1 fragments comprise an N-terminal 17-amino-acid amphipathic sequence (N17), followed by a variable-length polyQ tract, a 38-residue proline-rich region (PRR), and a 12-residue C-terminal segment [229]. Purified HTTex1 undergoes LLPS, primarily driven by the polyQ tract and PRR [229]. In vitro, HTTex1 forms three distinct phases: (1) M-phase, consisting of soluble monomers and oligomers; (2) S-phase, consisting of soluble aggregates; (3) F-phase, consisting of insoluble fibrillar aggregates [230]. In cells, mutant HTT forms dynamic, gel-like inclusions in which partial protein mobility persists, suggesting that large inclusions arise from random collisions of small aggregative particles [231]. Although several members of the HSPB family can prevent the aggregation of polyQ-HTTex1, including for example HSPB8 [232], HSPB7 is by far the most potent [227]. Its anti-aggregation activity is mediated by the intrinsically disordered NTD, which directly interacts with HTTex1 by binding the N17 and PRR regions [227, 233]. Full-length HSPB7 co-immunoprecipitates with polyQ-HTTex1, whereas deletion of the NTD abolishes both this interaction and its anti-aggregation activity [227, 233]. The NTD of HSPB7 is both necessary and sufficient for its potent anti-polyQ aggregation activity. Its NTD deletion abolishes binding to polyQ proteins and loss of aggregation suppression, while transferring the HSPB7 NTD onto another member of the family, such as HSPB1, confers anti-aggregation activity to the hybrid protein; by contrast, the CTD is dispensable for this function [233]. Direct evidence that HSPB7 is recruited to, or modulates, LLPS-driven HTT condensates is currently lacking. However, mutant HTT has been shown to undergo phase separation into condensates prior to irreversible aggregation [229]. Given that HSPB7 acts at early stages of amyloid formation, binding soluble protofibrils and preventing their progression into higher-order aggregates [227, 233], it is conceivable that HSPB7 may suppress polyQ aggregation by partitioning into polyQ-HTTex1 condensates via its NTD and interfering with condensate maturation, although direct experimental evidence for this mechanism is currently lacking. Alternatively, but not mutually exclusive, HSPB7 could target monomers and small oligomers before their incorporation into larger assemblies. Notably, ligand binding can alter saturation concentrations and shift phase boundaries through a mechanism known as polyphasic linkage [234]. By this principle, HSPB7 may preferentially bind M-phase polyQ-HTTex1 species (consisting of soluble monomers and oligomers), destabilizing both aggregation and phase separation by raising the concentration threshold required for LLPS. Such a mechanism would stabilize monomers and small oligomers, while suppressing the formation of larger aggregates and insoluble fibrillar phases [230]. Nevertheless, the potential role of HSPB7 in HTT phase separation remains to be elucidated.

### HSPB1, HSPB5 and HSPB7 and nuclear splicing speckles

Nuclear splicing speckles are dynamic, membraneless condensates enriched in spliceosomal components, including snRNPs and SR proteins such as SRSF2/SC35 [235, 236]. They act as reservoirs and regulatory hubs that coordinate pre-mRNA splicing [235]. Beyond splicing, recent evidence suggests that nuclear speckles influence gene expression [237], serving as gene-expression-inducing hubs that contain splicing factors that preferentially interact with specific gene sets that have unique sequence and splicing characteristics [237]. Perturbations in speckle composition or dynamics are associated with cancer, viral infection, and neurodegeneration [235, 237]. Here, we briefly summarize the main experimental evidence concerning HSPB1, HSPB5 and HSPB7 colocalization with and putative functions at the level of splicing speckles.

HSPB1 is mainly cytoplasmic under physiological conditions [238]. Upon stress conditions, such as heat shock [162, 239], ischemia/reoxygenation [240], or UV irradiation [241], a phosphorylated fraction of HSPB1 translocates to the nucleus and selectively accumulates in SC35-positive nuclear speckles [121, 122]. In addition, after heat shock, HSPB1 also colocalizes with nuclear speckles and the 20S proteasomal complexes, suggesting a potential role in PQC within splicing compartments [121]. Colocalization with nuclear speckles is transient and after stress recovery HSPB1 relocalizes to the cytoplasm [121]. The stress-induced localization of HSPB1 at splicing speckles may suggest that it may prevent the aggregation of SC35-resident proteins; however, interactors of HSPB1 at the level of nuclear splicing speckles have not yet been identified.

Similar to HSPB1, HSPB5 can be detected within nuclear splicing speckles, and this association is regulated by phosphorylation of HSPB5 NTD [123, 124, 127, 128]. Briefly, site-specific phosphorylation at S59 promotes nuclear import, while phosphorylation at S45 is essential for targeting HSPB5 to nuclear speckles, as demonstrated by mutagenesis experiments with phospho-mimicking and non-phosphorylatable mutants [125, 128]. Similar to HSPB1, it is unclear whether HSPB5 may bind to specific nuclear speckle-resident proteins to prevent their unfolding both under resting conditions and following heat shock. Interestingly, HSPB5 has also been detected in mitotic interchromatin granule clusters (MIGs) [125], which represent the mitotic counterparts of nuclear speckles [242]. MIGs arise from the redistribution of nuclear speckle components during the cell cycle. Notably, the recruitment of HSPB5 to these structures is strictly regulated by phosphorylation at S45, which is a key determinant for HSPB5 localization during the cell cycle (promoting its relocalization to nuclear speckles during interphase and MIGs during mitosis, respectively) [125].

Finally, HSPB7 localizes to nuclear speckles under physiological conditions and its enrichment within these condensates is further increased upon heat shock [126]. HSPB7 recruitment is strictly dependent on its NTD: deletion of this region or replacement with the NTD of HSPB1 abolishes speckle targeting, whereas fusion of the HSPB7 NTD to HSPB1 is sufficient to redirect it to speckles [126]. Despite its association with SC35 condensates, HSPB7 specific speckle-resident clients have not yet been identified.

Nuclear speckles undergo profound architectural rearrangements during stress responses and through mitotic progression [235, 236]. Together, these findings suggest that nuclear speckles could serve as hubs where, under physiological conditions, HSPBs may be involved in regulatory or structural functions by preventing the unfolding of specific clients. Upon stress or during mitosis, which induce speckle remodeling, these HSPBs could suppress aberrant protein-protein interactions, thereby preventing irreversible aggregation of speckle-resident proteins that could lead to partial speckle dysfunction.

### HSPB8

Also known as Hsp22, HSPB8 is a 21.6 kDa protein that is broadly expressed across multiple tissues, including brain, skeletal, and smooth muscle [243]. It forms a stable 2:1 stoichiometric complex with the HSP70 co-chaperone BAG3 (Bcl-2-associated Athanogene 3), which in turn binds to a single molecule of either Hsc70/HSPA8 or Hsp70/HSPA1A [136]. This complex has been characterized both in vitro [137, 244, 245] and in cells [136, 246]. Functionally, the HSPB8-BAG3 complex recruits HSP70 to direct misfolded proteins such as polyQ-expanded huntingtin, androgen receptor and ataxin 3, or SOD1 and ALS-associated dipeptide repeats to degradation via the autophagy-lysosome pathway [136, 247, 248]. Besides its pro-degradative activity, HSPB8 can also exert chaperone, anti-aggregation activity, as demonstrated in vitro, using recombinant HSPB8 alone, in the absence of BAG3 and the classical model substrates such as maltose-binding protein, citrate synthase, and rhodanese [48, 189]. In addition to these model substrates, in vitro HSPB8 also binds the disease-relevant proteins amyloid-β [143] and monomeric α-synuclein, neutralizing aggregation-prone regions and delaying fibril elongation and maturation into larger aggregates [249, 250]. HSPB8 appears to act on early, off-pathway aberrant species, preventing their conversion into stable and potentially toxic aggregates [48]. In addition, HSPB8 has been recently implicated in the prevention of the aggregation of RBPs and in the regulation of SG dynamics [116, 117].

**Fig. 7:**
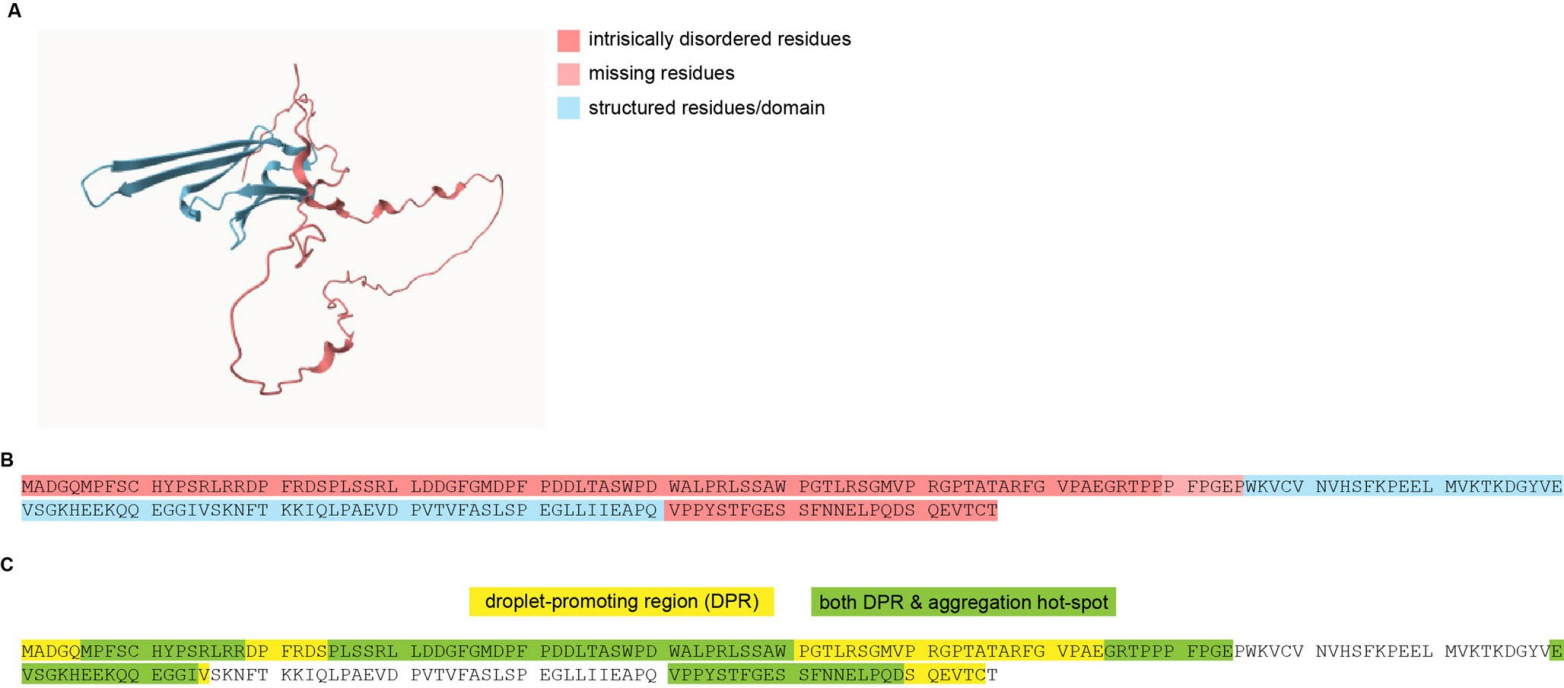
Predicted 3D structural model and LLPS potential of HSPB8. The 3D structural model of HSPB8 was generated using AlphaFold (**A**). (**B**) HSPB8 intrinsically disordered residues according to MobiDB (https://mobidb.org/). (**C**) HSPB8 droplet-promoting regions and aggregation hot-spot residues predicted by FuzDrop (https://fuzdrop.bio.unipd.it/predictor)

A structural and biophysical overview of HSPB8 is provided in Fig. 7.

#### HSPB8 and RBP condensates

As previously mentioned, SGs recruit aggregation-prone RBPs such as FUS and TDP-43, whose misfolding can lead to protein aggregation, SG solidification, and cellular toxicity [116, 117, 166]. HSPB8 is part of a surveillance system termed “granulostasis” that prevents the accumulation of misfolded proteins, including DRiPs, within SGs, thereby maintaining SGs dynamics and indirectly facilitating their disassembly [116]. During acute stress, HSPB8 transiently relocates from the BAG3-HSP70 complex to SGs, where it is thought to prevent the irreversible aggregation of RBPs and DRiPs [116]. At later stages of stress, SGs that have accumulated DRiPs and other misfolded proteins, recruit additional chaperones and co-chaperones, including BAG3, but also VCP, to promote their clearance [116, 175, 251]. Consistently, cells expressing either wild-type FUS or the ALS-linked FUS G156E mutant showed a delay of SG disassembly upon siRNA-mediated depletion of BAG3, which destabilizes HSPB8 leading to a drop in its expression levels [116]. Mechanistically, in vitro experiments showed that HSPB8 is recruited into FUS droplets to prevent the irreversible aggregation of FUS [117]. FUS undergoes LLPS through its LC/prion-like domain, while unfolding of the RRM promotes droplet aging and solidification [117]. The arginine-rich IDR of HSPB8 preferentially recognizes tyrosine-rich motifs in the FUS LC; this recognition positions the HSPB8 ACD in proximity to the FUS RRM, stabilizing it and reducing its unfolding, thereby preventing droplet aging and irreversible aggregation [117]. Of note, the neuropathy-associated K141E mutation in HSPB8 [252], which strongly reduces HSPB8 chaperone activity [48, 253], also decreased the interactions with FUS RRM1 and could not prevent FUS droplet solidification [117]. Whether in cells HSPB8 exerts a similar stabilizing effect on FUS, and potentially on other RBPs that are recruited inside SGs, is currently unknown.

## Yeast Hsp26 and Hsp42

**Fig. 8:**
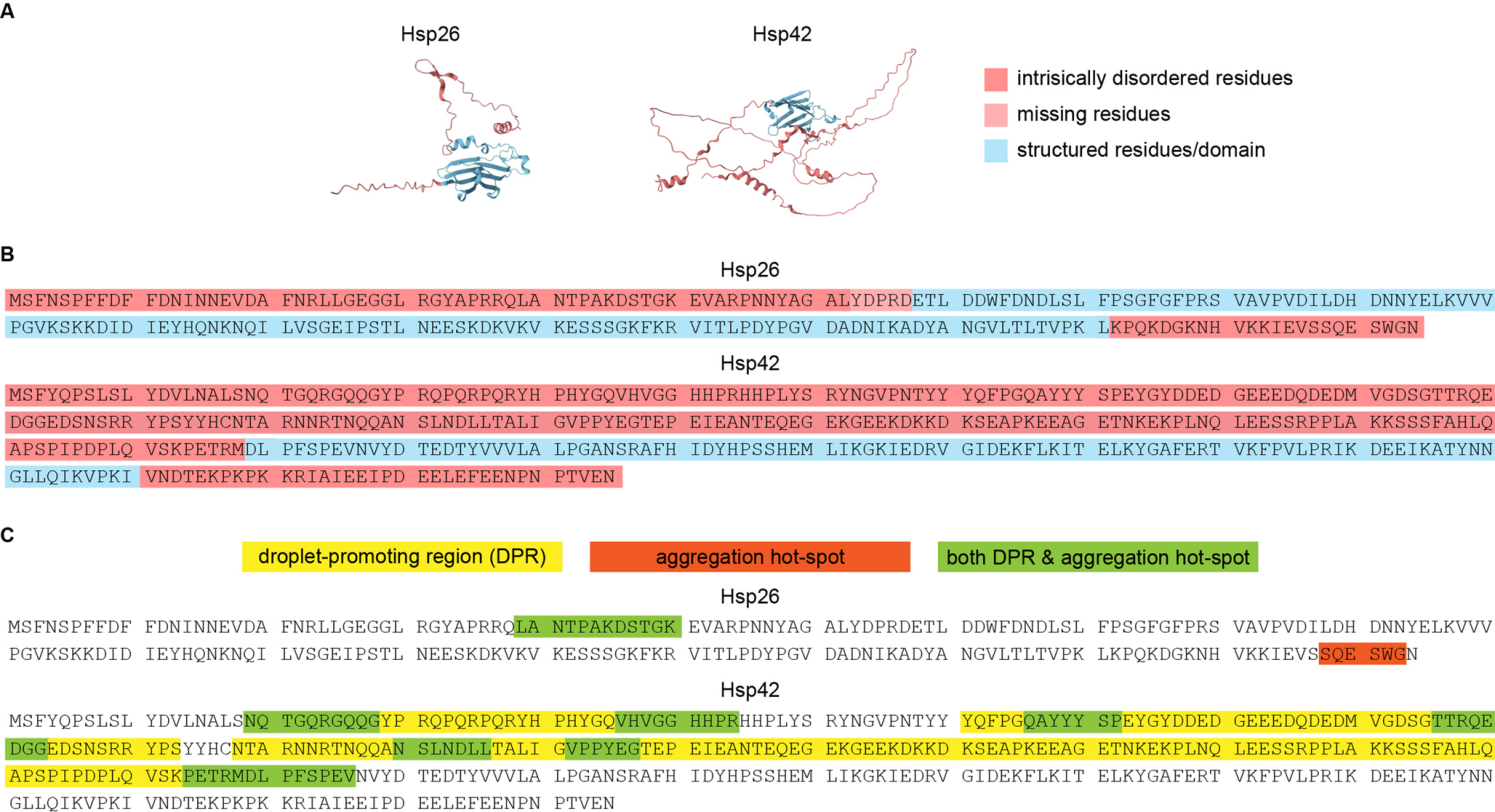
Predicted 3D structural model and LLPS potential of yeast Hsp26 and Hsp42. The 3D structural model of Hsp26 and Hsp42 was generated using AlphaFold (**A**). (**B**) Their intrinsically disordered residues were predicted using MobiDB (https://mobidb.org/). (**C**) Hsp26 and Hsp42 droplet-promoting regions and aggregation hot-spot residues predicted by FuzDrop (https://fuzdrop.bio.unipd.it/predictor)

The genome of Saccharomyces cerevisiae encodes two canonical sHSPs: Hsp26 and Hsp42 [254]. Both proteins harbor IDRs in their NTD, with Hsp42 additionally containing a PrLD that binds to misfolded proteins to promote their deposition at protein deposition sites [255]. Their AlphaFold-generated 3D structural model, their intrinsic disorder profiles, LLPS potential and droplet-promoting regions predicted by MobiDB and FuzDrop, respectively, are shown in Fig. 8. Although direct experimental evidence for phase separation of Hsp26 and Hsp42 in vitro is currently lacking, they colocalize with various stress-induced compartments in yeast and participate in spatial protein quality control, processes suggested to be regulated, at least in part, via phase separation [256–264]. Stress conditions such as heat shock, pH fluctuations, nutrient deprivation, or oxidative stress trigger the active and reversible sequestration of proteins into distinct compartments, including CytoQ (Cytosolic Quality-control compartment), IPOD (Insoluble Protein Deposit), JUNQ (Juxtanuclear Quality-control compartment), SPGs (Stationary-phase Granules), SGs, proteasome storage granules (PSGs), and quiescence granules. In only a few cases biophysical characterizations conclusively support classification of these structures as bona fide phase-separated condensates [265–269].

Concerning functionality, Hsp26 and Hsp42 contribute to an integrated proteostasis network, with distinct but complementary roles. Hsp26 functions primarily as a holdase, stabilizing early misfolded intermediates to facilitate their refolding by ATP-dependent chaperones and to prevent their premature coalescence into larger aggregates [42, 270, 271]. Hsp26 colocalizes with stress-induced assemblies but is not a primary driver of spatial sequestration [257, 263, 272]. In contrast, Hsp42 is a “sequestrase” or “aggregase” that promotes the sequestration of misfolded or aggregation-prone proteins into ER-associated CytoQ, to facilitate their subsequent clearance or refolding [256, 257, 264, 273]. The aggregase activity of Hsp42 relies on its NTE, which harbors both a PrLD and an IDR. This domain mediates oligomerization and multivalent interactions with misfolded substrates, facilitating CytoQ assembly [255, 257]. While CytoQ have gel-like properties and are sometimes referred to as aggregates or condensates, whether they form via LLPS remains unresolved and direct biophysical evidence is still limited [71, 273–278]. Here we summarize the published data reporting colocalization of Hsp26 and Hsp42 with different types of condensates.

### Hsp26 and Hsp42 in heat SGs

Upon heat shock, Hsp26 and Hsp42 are recruited to SGs [260–262]. In yeast, SGs can also be induced by starvation, oxidative stress, and stationary phase, but their assembly is often nucleated by misfolded proteins, unlike mammalian SGs [100, 262, 279, 280]. Under mild heat shock, SGs and misfolded protein aggregates assemble independently, whereas severe heat stress promotes nucleation of SGs by misfolded proteins, which then recruit Hsp42, Hsp26, and other chaperones [260–262]. The ATP-independent sHSPs are not essential for SG assembly or disassembly, and their recruitment may serve to target specific subsets of misfolded proteins; alternatively, their recruitment inside SGs could be a passive consequence of their IDRs [260, 266, 269, 281, 282]. Although in cells these sHSPs are dispensable for SG disassembly, in vitro studies showed that Hsp26 can prevent nucleation and sedimentation of the SG protein Pab1, suggesting a potential regulatory role in heat SG phase separation [269].

### Hsp26 and Hsp42 in Stationary Phase Granules (SPGs)

During chronological aging, Hsp42 promotes the relocalization of enzymes and regulatory proteins into cytosolic SPGs (Hsp42-SPGs), downregulating their activity and helping aged cells adapt to environmental stress [263, 283, 284]. Quiescence also leads to transient sequestration of epigenetic regulators, such as the nuclear histone deacetylase Hos2, into Hos2-SPGs, which recruit Hsp26 and Hsp42. Deletion of Hsp42, but not Hsp26, prevents Hos2-SPG formation [263]. Although SPGs display properties consistent with biomolecular condensates (lack of membranes, reversibility), their biophysical relationship to heat-induced SGs and LLPS remains unclear.

### Hsp26 and Hsp42 in Proteasome Storage Granules (PSGs) and IPODs

Under stress, proteasomes accumulate in PSGs, where ubiquitin chains act as scaffolds, promoting condensate formation and sequestering soluble ubiquitinated proteins to protect them from insoluble deposition [285]. PSGs may colocalize with Hsp42 and IPODs, insoluble aggregates near the vacuole primarily containing irreversibly aggregated amyloid and misfolded proteins [286–289]. Dysfunctional proteasome subunits retained in IPODs, along with Hsp42 and misfolded proteins, are targeted for autophagic clearance (proteaphagy) [258, 287, 290]. Thus, two distinct proteasome pools coexist under severe stress: functional proteasomes in PSGs, and misfolded proteasomes in IPODs with Hsp42 promoting autophagic clearance [289].

Overall, yeast sHSPs can colocalize with stress-induced condensates, rather than serving as primary scaffolds for their assembly, similar to what has been documented for mammalian HSPBs. Recruitment of sHSPs to SGs and other condensates may buffer misfolded proteins, slow gelation, and mitigate toxicity, supporting cellular proteostasis during stress and aging.

## Concluding remarks and future perspectives

In this review, we have summarized both earlier observations and recent experimental findings documenting the partitioning of sHSPs, primarily human HSPBs, into biomolecular condensates. Condensates are tunable, non-equilibrium assemblies whose composition, material properties and biological outcomes are continuously shaped by parameters such as concentration, interactions, PTMs and energy fluxes. Establishing causal links between condensate material states and biological function remains a central challenge. This review focused on human HSPBs, which can be recruited into pre-existing condensates, including SGs, p62 bodies and nuclear speckles. Within condensates, HSPBs prevent the irreversible aggregation of proteins (e.g., FUS, TDP-43), helping to preserve their liquid-like material properties. By competing with or diluting potentially aberrant strong protein-protein interactions, sHSPs/HSPBs may increase the local buffering capacity for unfolded or metastable proteins, effectively biasing condensates toward dynamic and reversible states. PTMs such as phosphorylation would allow these interactions to be tuned rapidly and reversibly, enabling stress-responsive recruitment and fine control over condensate behavior. In this way, sHSPs/HSPBs would not act as stoichiometric binders, but as regulators of the condensate interaction landscape, transiently stabilizing non-native conformations to provide a temporal window for active, ATP-dependent processes to remodel or disassemble the condensate. The ability of HSPBs to partition into condensates and modulate their dynamics is influenced by several factors, including their oligomeric state, phosphorylation status, and stoichiometric balance, as exemplified by the HSPB2-HSPB3 complex. The experimental data identify the disordered NTD and CTD, as well as phosphorylation-regulated oligomeric transitions, as key determinants of HSPB recruitment into condensates and chaperone activity. Yet, how different HSPBs discriminate between distinct types of condensates or respond to stress-specific versus constitutive assemblies, remains poorly understood. Key open questions include the relative roles of sequence motifs, charge patterns, oligomeric state, and local abundance in determining selective recruitment and client engagement. Resolving these questions will require structural studies of IDRs and oligomeric transitions, alongside functional experiments linking HSPB partitioning to enzymatic activity, signaling and condensate remodeling. Addressing these questions will require integrated approaches combining high-resolution structural biology, quantitative biophysics and functional cell biology. Moreover, understanding how sHSP/HSPB recruitment interfaces with energy-consuming remodeling processes will be critical to define their role in shaping the dynamic, non-equilibrium nature of condensates. HSPB-mediated modulation of condensate properties also has broad implications for proteostasis. Many condensates serve as intermediates in degradation pathways, including polyubiquitin-rich p62 bodies, proteasome storage granules, and autophagosome nucleation sites. By transiently binding clients and influencing condensate material properties, HSPBs may enhance the efficiency of client recognition, sequestration, and clearance, buffering proteotoxic stress and maintaining cellular function. Perturbations in this system, such as disease-associated HSPB mutations (e.g., HSPB3-R116P, HSPB8-K141E), can impair condensate dynamics, leading to pathological protein accumulation and neuromuscular degeneration.

In summary, beyond acting as first-line defenders against protein aggregation, sHSPs/HSPBs may function as modulators of the condensate interaction landscape, constraining transitions to pathological states while promoting reversible and functional condensate behavior. 

## Data Availability

Data sharing is not applicable to this article as no new data were created.

## Declarations

The authors employed AI based language support for text refinement; all scientific content was conceived, analyzed, and validated by the authors.
